# Nickel‐Regulated Composite Cathode with Balanced Triple Conductivity for Proton‐Conducting Solid Oxide Fuel Cells

**DOI:** 10.1002/advs.202304555

**Published:** 2023-10-28

**Authors:** Hua Tong, Wenjing Hu, Min Fu, Chunli Yang, Zetian Tao

**Affiliations:** ^1^ School of Resources, Environment and Safety Engineering University of South China Hengyang Hunan 421001 China; ^2^ College of Materials Science and Engineering Xi'an University of Architecture and Technology Xi'an 710043 China

**Keywords:** composite cathode, proton conductor, solid oxide fuel cells, triple conductivity

## Abstract

Proton‐conducting solid oxide fuel cells (H‐SOFCs) have the potential to be a promising technology for energy conversion and storage. To achieve high chemical compatibility and catalytic activity, nickel‐doped barium ferrate with triple conducting ability is developed as cathodes for H‐SOFCs, presenting an impressive electrochemical performance at intermediate temperatures. The cell performance with the optimized BaCe_0.26_Ni_0.1_Fe_0.64_O_3_–_δ_ (BCNF10) composite cathode reaches an outstanding performance of 1.04 W cm^−2^ at 600 °C. The high electrocatalytic capacity of the nickel‐doped barium ferrate cathode can be attributed to its significant proton conductivity which is confirmed through hydrogen permeation experiments. Density functional theory (DFT) calculations are further conducted to reveal that the presence of nickel can enhance processes of hydration formation and proton migration, leading to improve proton conductivity and electro‐catalytic activity.

## Graphical abstract

Doping of Nickel can regulates the composition of the composite cathode with heterojunction structure to reach a balance of triple conductivity which is crucial for catalytically activity of cathode materials. Based on the regulated cathode, an outstanding electrochemical performance of 1.04 W cm^−2^ at 600 °C is achieved which may be one of the highest performance for cobalt‐free cathodes.

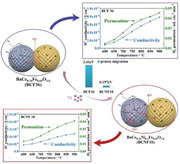

## 1 Introduction

Compared to other fuel cell types, proton‐conducting solid oxide fuel cells (H‐SOFCs) operate at lower temperatures and require less activation energy, allowing them to achieve more efficiency and with higher power density between 500 and 700 °C.^[^ ^1^ ^]^ Among typical proton conductors, BaCe_0.7_Zr_0.1_Y_0.2_O_3_ (BZCY) is an ideal electrolyte in H‐SOFCs due to the high mobility of protons, providing both high proton conductivity and excellent chemical stability.^[^ ^2^ ^]^ Despite many advantages of H‐SOFCs, their large‐scale commercial utilization is still limited by the challenge of developing highly active cathodes that can operate efficiently at intermediate temperatures.^[^ ^1^, ^3^ ^]^ In early research, some cobalt‐based cathodes have been widely used as cathodes for their high catalytic activity toward oxygen reduction reaction which is crucial to the cathode reaction performance. In addition to traditional materials such as La_0.6_Sr_0.4_Co_0.2_Fe_0.8_O_3–δ_ (LSCF) and Ba_0.5_Sr_0.5_Co_0.8_Fe_0.2_O_3–δ_ (BSCF), other cobalt‐based oxides with high mixed conductivity and robust perovskite crystal structure are also investigated as H‐SOFCs cathodes to accelerate oxygen reduction reaction (ORR), which is relevant to the energy conversion technologies’ efficiency.^[^ ^4^ ^]^ However, the susceptibility to operation degradation as well as the high cost of cobalt restricts the lifetime and large‐scale deployment of cobalt cathodes.^[^ ^5^ ^]^

To address these challenges, researchers are exploring inexpensive alternative cathode materials, offering similar or better performance and greater stability under fuel cell operating conditions. At the cathode side of H‐SOFCs, protons migrated through the electrolyte layer and reacted with the oxygen ions that have adsorbed onto the cathode surface to form water. The concurrent conductivity of oxygen ions, protons, and electrons in a cathode material would not only facilitate the cathode reaction but also extend the reaction area across the entirety of the electrode.^[^ ^6^ ^]^ Ongoing research is focused on developing new triple‐conducting cathode materials with improved properties, as well as optimizing their operating conditions to maximize their performance in fuel cells.

One potential cathode material for H‐SOFCs that has been extensively studied is single phase BaFeO_3_ (BFO),^[^ ^7^ ^]^ considering that transition metal Fe has variable oxidation and spin states, contributing to high catalytic activity in air environment^[^ ^8^ ^]^ and a range of promising properties, so that BFO becomes an attractive candidate cathode for use in H‐SOFCs. By partially substituting different ions into the BFO lattice, the material's properties can be modified to improve its conductivity and electrochemical activity, ultimately leading to better overall cathode performance.^[^ ^5^, ^9^ ^]^ Ce‐doped BFO was found to decompose into two uniformly dispersed perovskite oxides at high temperatures and exhibited intrinsic triple‐conducting behavior which can facilitate the cathode reaction processes at various sites throughout the cathode.^[^ ^6^, ^10^ ^]^ Besides, nickel ions have been commonly adopted as an additional dopant due to their relatively lower oxidation state and larger ionic radius, which can improve its catalytic activity for ORR as well as the durability of H‐SOFCs.^[^ ^11^ ^]^

Recently, our group researched BaCe_x_Fe_1–x_O_3–δ_ (BCF) cathodes, and excellent electrochemical performance is achieved by tailoring the Ce/Fe ratios to balance the synergistic effect of self‐assembled composites.^[^ ^12^ ^]^ Despite exhibiting high oxygen catalytic activity and electrochemical performance, BCF cathodes still encounter challenges related to their relatively low electronic conductivity and lack of clarity regarding their triple‐conducting mechanism. In this article, Ni‐doped BaCe_0.36_Fe_0.64_O_3–δ_ (BCF36) is reported and the internal mechanism of superior cathode catalytic activity is carefully studied. The single cell with cathode displays a record maximum power density at 600 °C. Replacing Ni in the B sites of BCF36 with the appropriate ratio can significantly lower the migration barrier of proton conduction and hydration as well as oxygen vacancy formation, according to both our experimental study and density functional theory (DFT) calculations.

## 2 Results and Discussion

### 2.1 Crystal Structure and Characterization

**Figure 1:**
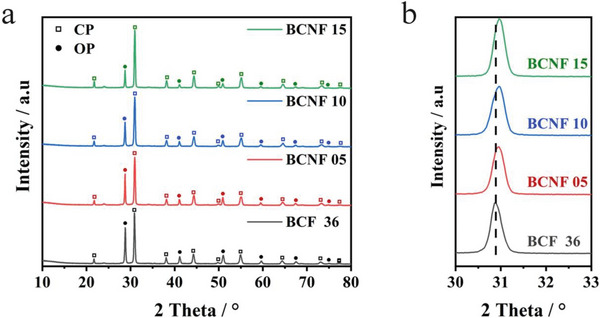
XRD patterns of BCF 36 and BCNF series oxides a) 2θ from 10° to 80°. b) 2θ from 30° to 33°.

The XRD patterns of the BCNF series powders, which were prepared using a wet chemical route, were examined to determine their chemical structures. **Figure** **1** presents the results of the analysis. Specifically, Figure 1a illustrates the XRD patterns of the Ni‐doped BCF36 samples with varying Ni content. The samples exhibit the same lattice structures as BCF36, which includes two different types of perovskite oxides: cubic perovskite (CP) and orthorhombic perovskite (OP) structures. Additionally, when substituting 5 – 15% of Ni, the structural phases remain unchanged, but the main diffraction peak of the CP phase at 2θ = 30.74° shifts to a higher angle, as observed in Figure 1b. Conversely, the peaks associated with the OP phase show no significant changes, indicating successful doping of Ni into the lattice of BaFeO_3_. Table S1 (Supporting Information) presents the lattice parameter of the CP phase, which decreases from 4.092 to 4.08 Å with increasing Ni doping content. This can be attributed to the smaller ionic radius of Ni^3+^ (Ni^3+^ = 0.560 Å) compared to Fe^3+^ (Fe^3+^ = 0.64 Å), resulting in a compression of the lattice structure as more Ni is incorporated.^[^ ^13^ ^]^ The XRD Rietveld refinement results for BaCe_0.36‐x_Ni_x_Fe_0.64_O_3__‐__δ_ (BCNFx, x=0,0.05,0.1,0.15), as shown in Figure S1 (Supporting Information), provide important information about the crystal structures present in the material. For BCNF10, the refinement reveals that the composition consists of CP phase (79.04 wt.%) and OP phase (20.96 wt.%) with a reasonable reliability fitting factor of 4.956. Similarly, BCNF05 has a composition of 75.07 wt.% CP phase and 24.92 wt.% OP phase, while BCNF15 contains 85.55 wt.% CP phase and 14.45 wt.% OP phase, as determined by XRD refinement. These refinement results provide reverse verification and support the conclusions drawn earlier regarding the crystal structures and their respective compositions in the BCNFx material. Meanwhile, Figure S2 (Supporting Information) provides a clear insight into the doping mechanism of Ni, as determined through DFT calculations. The results demonstrate that the binding energy of Ni doping into BaFeO_3_ is −271.49 eV, which is much lower than that of Ni doping into BaCeO_3_ (Binding energy = −145.3 eV), further suggesting that Ni has a greater tendency to enter into the lattice of BaFeO_3_.

**Figure 2:**
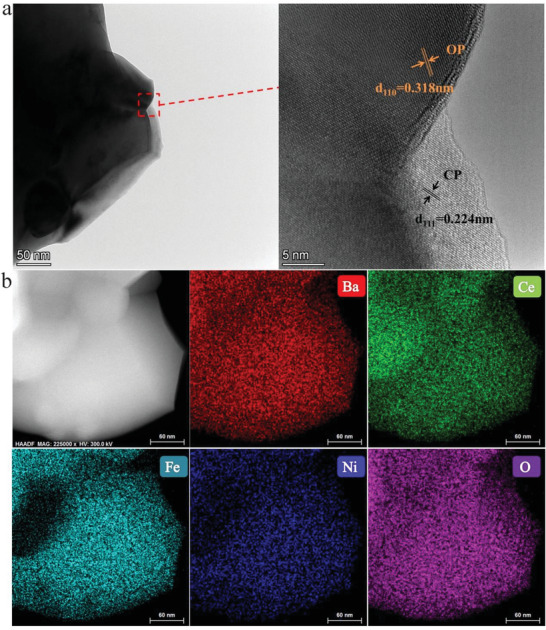
Self‐assembled heterogeneous junction structure and elemental distribution a) TEM and corresponding fast fourier transform (FFT) images of the BCNF10 oxide. b) EDX‐mapping result of the BCNF10 oxide.

**Figure** **2**a presents a transmission electron microscopy (**TEM**) image of a synthesized BCNF10 grain with a size of ≈ 650 nm, displaying a smooth nanoparticle surface. Afterward, high‐resolution transmission electron microscopy (HR‐TEM) technology is employed to examine the micromorphology of BCNF10, revealing that the lattice spacing values of pure CP and OP are 2.236 Å (111) and 3.18 Å (110) respectively. Interestingly, it is observed that the distance of the (111) diffraction plane appeared narrower compared to the values reported in the PDF card (PDF•#75‐0426), indirectly verifying that Ni is doped into the BFO lattice, causing lattice contraction of the CP phase. Figure 2b depicts the X‐ray energy dispersive spectrum (EDX) mapping of the BCNF10 particle, illustrating that Ba as well as O was uniformly distributed in the selected grain while Ce and Fe were separately distributed within the particles. Moreover, the distribution area of Ni is consistent with that of Fe, providing further evidence of Ni doping into the lattice of BaFeO_3_.

### 2.2 Kinetic Properties and Valence Distribution

**Figure 3:**
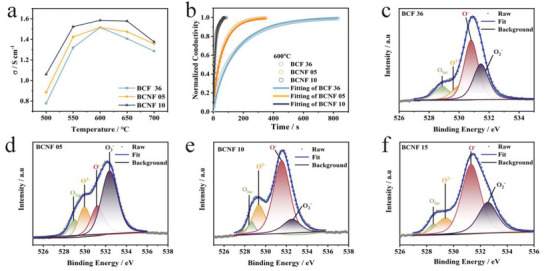
Electron/oxygen ion conductivities and fitted graphs of O1s spectra a) Electrical conductivity of BCF36, BCNF05, and BCNF10 from 500 to 700 °C at ambient air. b)ECR profiles at 600 °C with oxygen partial pressure switching from 0.21 to 0.5 atm of BCF36, BCNF05, and BCNF10. XPS O1s spectra at room temperature for c) BCF36 sample, d) BCNF05 sample, e) BCNF10 sample and f) BCNF15 sample.

The electrical conductivity relaxation (ECR) technique is used to investigate the kinetic properties of BCNF materials, and the results are presented in **Figure** **3**. In Figure 3a, it can be observed that the conductivities of all the synthesized materials initially increase and reach a maximum value at 600 °C, after which they start to decrease. Below 600 °C, these materials exhibit typical semiconductor behavior, where electron transport occurs through hole hopping along the Fe‐O‐Fe chains and is thermally activated. However, at temperatures above 600 °C, the conductivities decrease with increasing temperature, indicating a transition to metallic conductivity in the BCNF series. This behavior can be attributed to the loss of oxygen, the reduction of Fe at high temperatures, the decrease in the number of electron holes, and changes in the Fe‐O‐Fe charge transfer mechanism.^[^ ^14^ ^]^ In addition, the electrical conductivity of BCNF at ambient air is higher than that of pure BCF due to the increase in the concentration of oxygen vacancies in the lattice as a result of Ni doping, which creates more charge carriers and improves charge transfer during the cathode process (Figure 3a). The relaxation time is plotted on the horizontal axis, representing the time it takes for the sample to reach a new equilibrium after the oxygen partial pressure is changed from 0.21 atm to 0.5 atm. The electrical conductivities of BCF36 and BCNF series powder at different temperatures are normalized and fitted to surface‐controlled equilibration kinetic. The surface exchange coefficient (K_ef_) and diffusion coefficient (D_ef_) are determined and summarized in Table S2 (Supporting Information).^[^ ^15^ ^]^ In Figure 3b, the conductivity relaxation processes for BCNF series materials at 600 °C are depicted, and ECR response curves for BCF36, BCNF05, and BCNF10 at 700 °C are displayed in Figure S3 (Supporting Information). The K_ef_ and D_ef_ values for BCF36 are determined to be 8×10^−4^ and 3.8×10^−5^ cm^2^ s^−1^, respectively, while for BCNF10, the corresponding values are found to be 9×10^−3^ and 7.5×10^−4^ cm^2^ s^−1^. K_ef_ represents the rate of oxygen adsorption and dissociation, while D_ef_ is a key factor in oxygen ion diffusion inside the cathode. The values of K_ef_ and D_ef_ are important for reducing the polarization resistance of the cathode and improving its electrochemical performance.^[^ ^16^ ^]^ Based on these results, it can be concluded that BCNF10 has a better electrochemical performance in terms of oxygen ion transmission.

To clarify the detailed chemical states of BCNF series powders, X‐ray photoelectron spectroscopy (XPS) analysis of O1s are tested. As shown in Figure 3c–f, the O1s spectra showing the broad doublet peaks in all samples were analyzed by fitting them using four components, which corresponded to different oxygen species. These components included lattice oxygen (OL) and chemisorbed oxygen species (OC) which were composed of O^−^, O^2−^, and O_2_^−^. The processes of adsorption, dissociation, and migration of oxygen can be described as follows:

(1) $$O_{2\left(g\right)}\to O_{2\left(\mathrm{ads}\right)}\to O_{2}^{-}↔2O_{\left(\mathrm{bulk}\right)}^{-}\to O_{\left(\mathrm{bulk}\right)}^{2-}\to O_{\left(\mathrm{TPB}\right)}^{2-}$$

The ratio of chemisorbed oxygen species (OC) to lattice oxygen (OL) critically influences the ORR activity by facilitating the migration of chemisorbed oxygen to the cathode, where it reacts with protons,^[^ ^17^ ^]^ thereby accelerating oxygen kinetics.^[^ ^11c^ ^]^ Table S3 (Supporting Information) indicates that BCNF10 has the highest OC/OL ratio. Considering the result that the ratio of chemisorbed oxygen species (OC) to lattice oxygen (OL) is related to the oxygen vacancy,^[^ ^18^ ^]^ the subsequent calculation of the oxygen vacancy formation energy is conducted to further understand the reasons behind the increased formation of oxygen vacancies, suggesting that BCNF10 could perform more oxygen vacancies, exhibit the highest ORR activity, and obtain the superior cathode performance for H‐SOFCs.

### 2.3 Performance of PCFCs with BCNFx Cathodes

**Figure 4:**
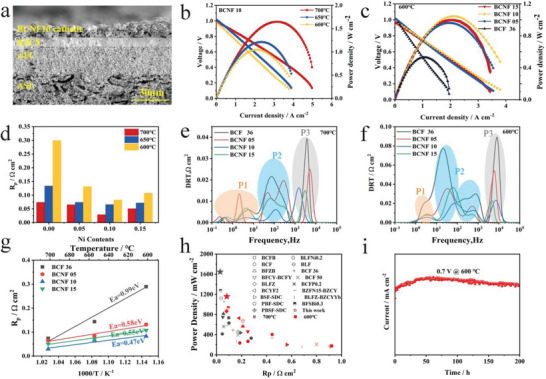
Cross‐sectional images of fuel call and comparisons in electrochemical performance and electrical impedance with various cathodes a) SEM overview of the BCNF10 powders. b) I – V, I – P curves of the single cells with the configurations of BCNF10 at 600 – 700 °C. c) MPDs comparison of the single cell with the BCF36, BCNF05, BCNF10, BCNF15 cathode at 600 °C. d) Dependence of the Rp for BCNF cathodes with different Ni contents at 600 – 700 °C. A comparison of DRT plots of BCF36, BCNF05, BCNF10, BCNF15 cathodes at e) 700 °C and f) 600 °C. g)Arrhenius plots of polarization resistances for BCF36, BCNF05, BCNF10, and BCNF15 cathode. h) MPDs comparison of BCNF10 with different cathodes. i) Operational stability of the single cell with BCNF10 as cathode under 3 vol% H_2_O‐H_2_ atmospheres among 200 h.

**Figure** **4**a shows the microstructure morphology with a porous anode supporting layer (ASL, Ni‐BZCY, ≈600 µm thick), an anode functional layer (AFL, Ni‐BZCY, ≈25 µm thick), a flat and dense electrolyte (BZCY, ≈9 µm thick), and a porous cathode (BCNF10, ≈10 µm thick). Additionally, Figure S4 (Supporting Information) presents scanning electron microscopy (SEM) images of BCF36, BCNF05, BCNF10, and BCNF15. A comparative analysis reveals that all four samples possess a similar electrolyte thickness, indicating that the variation in cell performance primarily arises from the differences in cathode materials. In order to investigate the influence of various Ni doping levels, the electrochemical performance was evaluated by subjecting the cell to wet (3 vol% H_2_O) hydrogen at the anode while the cathode was exposed to ambient air. The current‐voltage‐power (I‐V‐P) curves of the BCNF10 cathode with a BZCY electrolyte are measured and presented in Figure 4b. The values of open circuit voltage (OCV) are 0.988, 1.019, and 1.051 V at 700, 650, and 600 °C, respectively. These values are consistent with the SEM results and provide further confirmation of the dense nature of the electrolyte layer. As shown in Figure 4b, the maximum power densities (MPDs) of the BCNF10 cell are 1.64, 1.21, and 1.04 W cm^−2^ at 700, 650, and 600 °C, respectively. Figure S5a–d (Supporting Information) depict the power density versus current density curves for H‐SOFCs using BCNFx cathodes from 700 to 600 °C. Figure 4c presents the MPDs of BCNFx at 600 °C, aiming to directly observe the enhancement in electrochemical performance resulting from Ni doping. As can be seen from the Figure 4c, the MPDs of BCNF05, BCNF10, and BCNF15 are 0.96, 1.04, and 1.00 W cm^−2^, which are ≈1.81, 1.96, and 1.88 times higher than that of BCF36 (0.53 W cm^−2^). In addition, Figure 4 h has been generated by referring to previous literature research, which is consistent with Table S4 (Supporting Information), illustrating a comparison of the highest performance achieved at 600 and 700 °C when utilizing BaFeO_3_‐based perovskite materials as cathodes in H‐SOFC. In contrast, the results obtained in this study demonstrate that BCNF10 exhibits the highest catalytic activity, regardless of the temperature range between 600 and 700 °C. The typical electrochemical impedance spectra (EIS) spectra of the cell with BCNFx cathodes are measured under OCV conditions at temperature ranging from 700 to 600 °C, allowing for the evaluation of total resistance and polarization resistance (Rp) (Figure S6a–d, Supporting Information). For comparison, the bar chart in Figure 4d depicts the polarization resistances (R_p_) of BCNFx (x = 0, 0.05, 0.1, 0.15) cathode tested at various temperatures. It is evident from observation that BCNF10 exhibits the lowest Rp values, measuring 0.028, 0.065, and 0.082 Ω cm^2^ at 700, 650, and 600 °C, respectively. Meanwhile, the temperature dependence of R_P_ for each cathode is shown in the Arrhenius plot (Figure 4 g), revealing that the substitution of Ni at the Fe site in BCF36 leads to a reduction in the activation energy (Ea), with BCNF10 exhibiting the lowest Ea among the four materials.

In addition, the distribution of relaxation times (DRT) analysis is performed to identify the sub‐steps involved in the electrode reaction processes, allowing for the characterization of different relaxation processes and providing valuable information about the polarization resistance. As shown in Figure 4f and e, the DRT analysis reveals the presence of four or five distinct peaks, labeled as P1‐P3. In the low‐frequency range, P1 is possibly ascribed to the process of oxygen adsorption and dissociation on the electrode surface. The middle‐frequency P2 is closely associated with surface diffusion and oxygen ion bulk diffusion toward the triple phase boundaries (TPBs). The high‐frequency peak, P3, is influenced by the electrochemical reactions occurring at the cathode and anode, including charge transfer processes.^[^ ^19^ ^]^ In the diagram, at the temperature of 600 °C, there is no obvious change in the low‐frequency region except BCF36. However, the area of P2 of BCNF10 is the smallest within the frequency range of 10–1000 Hz, which suggests that the surface diffusion and oxygen ion bulk diffusion processes occur at the highest rate in BCNF10. The enhanced conductivity and efficient oxygen species diffusion rate of BCNF10 make it favorable for electrode reactions. Furthermore, the values of the P3 peak for BCNF10 and BCNF15 are similar and lower than those of the other materials. Additionally, when comparing the results at 600 and 700 °C, it can be observed that the DRT curve at 600 °C slightly shifts toward higher frequencies. The DRT intensity of BCNFx at 700 °C is lower than that at 600 °C, indicating that the electrode reaction rate at 700 °C is significantly faster. Moreover, the disappearance of peaks in the 0.1–1 Hz range at 600 °C suggests that the corresponding reaction process becomes the non‐rate‐limiting step. Based on these findings, it can be concluded that increasing the operating temperature can accelerate the rate of electrode reactions, and BCNF10 demonstrates great potential as a cathode material.

During the 200 – hour testing period, the long‐term electrochemical stability of the single cell with BCNF10 cathode was evaluated. Figure 4i displays the current density of the cell as a function of the given voltage of 0.7 V, operating at a temperature of 600 °C with H_2_ as the fuel. The results indicate that the current density initially increases gradually from 700 mA cm^−2^ to 900 mA cm^−2^ within the first 50 h of testing.^[^ ^20^ ^]^ Subsequently, over the next 150 h, no significant degradation is observed, demonstrating the stable structure and excellent thermal compatibility of this single cell.

### 2.4 Hydrogen Permeation and DFT Calculations

To gain deeper insights into the electronic structure and proton migration properties of Ni‐doped BCF36, a computational analysis with density functional theory (DFT) was conducted. Figure S7 (Supporting Information) exhibits the optimized structures of BaFeO_3_ (BFO) and BaNi_0.125_Fe_0.875_O_3_ (BNFO). To investigate the effects of Ni doping, the oxygen formation energy was calculated according to the following equation for BNFO and BFO.

(2) $$E_{\mathrm{formation}}=E_{\mathrm{defect}}+\frac{1}{2}E_{O_{2}}-E_{\mathrm{perfect}}$$

Both materials are more prone to form vacancies in the Fe‐O‐Ni position, as illustrated in Figure S8 (Supporting Information). Since there are 24 oxygen atoms in the structure, it is necessary to calculate the oxygen vacancy energy at 24 positions. The calculated oxygen formation energy for BNFO was found to be only 1.7286 eV, which is lower than that of BFO (2.2449 eV), indicating that the oxygen vacancy could be more easily formed in BNFO compared to BFO, which is consistent with the XPS results.

In addition to the formation of oxygen vacancies, it is important to consider the protonation ability or hydration of cathode materials in H‐SOFCs. This is because oxygen ions react with protons that are transferred from the anode, leading to the formation of water at the cathode side. Therefore, the ability of the cathode material to facilitate proton transfer and hydration reactions is crucial for the efficient operation of H‐SOFCs. The hydration energy is defined as the following equation^[^ ^5^, ^21^ ^]^

(3) $$\Delta \;E_{\mathrm{hy}}=2E_{\mathrm{OH}}-E_{V_{O}^{••}}-E_{H_{2}O}$$

**Figure 5:**
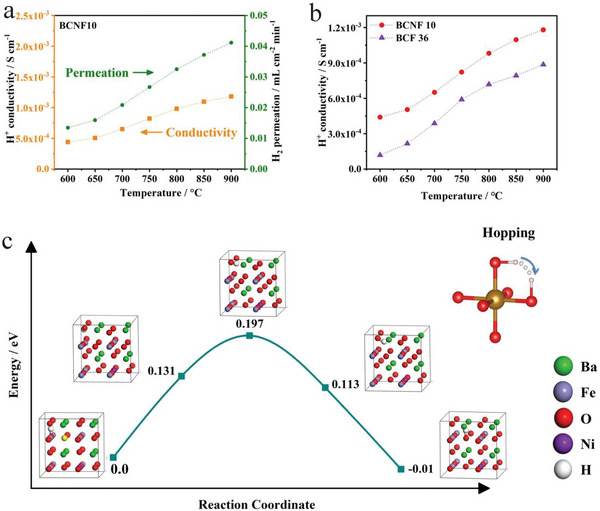
Proton conductivity and DFT calculations for proton migration a) the calculated proton conductivity and hydrogen permeability of BCNF10. b) Comparison of proton conductivities for BCF36 and BCNF10 between 600 and 900 °C. c) Calculated energy profiles for proton migration.

The hydration ability of BNFO was found to be significantly superior to that of BFO, as evidenced by its lower hydration energy of 0.699 eV compared to BFO's higher hydration energy of 2.1028 eV. This suggests that BNFO has a stronger affinity for water molecules and can undergo hydration more readily than BFO, indicating that the doping of the high basicity element Ni can enhance the hydration ability and promote the formation of protons. Another critical factor affecting the performance of cathode materials for H‐SOFCs is the ability of proton transportation, specifically the migration of protons between oxygen atoms. The proton migration energies and structure at the initial state, transition state, and final state are presented in **Figure** **5**c. The initial state was optimized by bonding a hydrogen atom with the oxygen atom between transition metal atoms and the final state was optimized by bonding a hydrogen atom with the oxygen atom neighboring the initial state H‐connected oxygen. The lower energy barrier for proton migration of BNFO (0.197 eV) compared to BFO oxides (2.42 eV) indicates that Ni‐doping can significantly reduce the energy barriers for proton migration in BNFO, leading to enhanced proton conductivity and improved overall performance in H‐SOFCs.

The influence of Ni doping on proton migration was visually observed through hydrogen permeation measurement. The proton conductivity of the materials could be obtained by the following Wagner equation:

(4) $$\sigma_{H^{+}}=\frac{J_{H_{2}}\frac{4F^{2}L}{RT}}{\ln\frac{P_{H_{2},\mathrm{supp}}}{P_{H_{2},\mathrm{perm}}}}$$

where $\sigma_{H^{+}}$ is the proton conductivity, $J_{H_{2}}$ represents the H_2_ permeation flux and *F* is the Faraday coefficient which is 96 485.33 C mol^−1^, *L* represents the thicknesses of BCF36 (0.86 mm) and BCNF10 (0.63 mm). The variable *R* represents the ideal gas constant, which has a value of 8.314 J (mol K)^−1^. *T* represents the operating temperature in Kelvin. $P_{H_{2},\mathrm{supp}}$ and $P_{H_{2},\mathrm{perm}}$ represent the hydrogen partial pressure on the hydrogen supply side and the permeate side, respectively.

In Figure 5a, the temperature dependence of hydrogen permeation through BCNF10 cermet membranes was calculated and plotted. It can be observed that the BCNF cermet exhibited the highest flux, reaching 0.0 4115 mL cm^−2^ min^−1^ at a temperature of 900 °C. In Figure 5b, it is evident that BCNF10 exhibits significantly higher proton conductivity compared to BCF36. At a temperature of 600 °C, the calculated proton conductivities for BCNF10 and BCF36 are 4.402×10^−4^ and 1.181×10^−4^ S cm^−1^, respectively, demonstrating that the incorporation of 10 mol% Ni in the cermet composition enhances the proton conductivity. The improved proton conductivity in BCNF10 facilitates migration of protons and extends the TPB to the entire cathode areas (Figure S9, Supporting Information). Consequently, the optimization of TPBs could efficiently reduce polarization resistance and enhance cell performance.

## 3 Conclusion

A novel cobalt‐free cathode material, BaCe_0.36‐x_Ni_x_Fe_0.64_O_3‐δ_ (x = 0, 0.05, 0.1, 0.15, BCNFx), was developed and utilized in proton‐conducting solid oxide fuel cells (SOFCs). The introduction of partial Ni doping in the cathode resulted in improved hydrogen permeation capability and reduced oxygen vacancy formation energy. Additionally, density functional theory (DFT) calculations confirmed the enhanced transmission rate of protons and hydration ability of the BCNFx cathodes. In terms of electrochemical performance, the cell using BaCe_0.26_Ni_0.1_Fe_0.64_O_3_ (BCNF10) as the cathode exhibited remarkable achievements, reaching a record maximum power density (MPD) of 1044 mW cm^−2^ at 600 °C. This highlights the successful design strategy of balancing triple conductivity, leading to highly efficient proton‐conducting SOFCs.

## 4 Experimental Section

### Powder Synthesis

The BaCe_0.36‐x_Ni_x_Fe_0.64_O_3‐δ_ (x = 0, 0.05, 0.1, 0.15, BCNFx) powders were synthesized by the sol‐gel method. The raw materials used for cathode BCNFx powders were BaCO_3_, Ni(NO_3_)_2_·6H_2_O, Fe(NO_3_)_3_·9H_2_O and Ce(NO_3_)_3_·6H_2_O. First, the suitable weights of the the initial components were determined with the molecular formula's stoichiometric ratio. Then citric acid, which has a 1.5:1 molar ratio to metal ions, was added as a complexing agent after these raw materials had been dissolved in the diluted nitric acid solution, and ammonia was used to modify the pH of the solution to 7–8. After allowing the solution to spontaneously burn at 80 °C, precursor powders were produced, further calcined for 3 h at 1000 °C. BaCO_3_, Zr(NO_3_)_4_·5H_2_O, Ce(NO_3_)_3_·6H_2_O and Y(NO_3_)_3_·6H_2_O were served as the raw materials for electrolyte BaZr_0.1_Ce_0.7_Y_0.2_O_3‐δ_ (BZCY) powders in the same way. Subsequently, after being calcined in a Muffle furnace, the BZCY powder along with nickel oxide and starch (weight ratio of 40:60:20) was homogenized by ball milling for 24 h to obtain anode powder NiO‐BZCY.

### Single Cells Fabrication

The half cells were prepared by a dry‐pressing method. Initially, a mixture consisting of NiO, BZCY, and corn starch in a weight ratio of 60%:40%:20% was pre‐pressed at 200 MPa to form the substrate. Next, the anode functional layer and BZCY electrolyte powders were separately pressed onto this substrate under 300 MPa. The assembly was then sintered at 1350 °C for 5 h. The cathode slurry was fabricated by grinding cathode powders and mixing it with terpineol for 2 h. The single cell configuration, NiO‐BZCY/BZCY/BCNFx, was achieved by applying the cathode slurries onto the electrolyte layer with an effective area of 0.237 cm^2^ and co‐firing them at 1000 °C for 3 h.

### Materials Characterization

X‐ray diffraction (XRD) analysis was employed to determine the purity of the synthesized powders and investigate their phase composition. The technology of X‐ray photoelectron spectroscopy (XPS) was applied to investigate different valence states of the elements. Scanning electron microscopy (SEM) was used to display the microstructure of single cells and transmission electron microscopy was performed to explore the composition of the cathode materials. The measurement of hydrogen permeation flux was conducted using a custom‐built apparatus. The sintered BCNFx pellets were carefully prepared by polishing both sides with SiC sandpaper to achieve a flat and parallel surface. Each pellet had a uniform thickness of ≈0.6 mm to ensure consistent and comparable results. To create a reliable seal, the pellets were securely mounted on an alumina tube using a glass ring sealant and subjected to a sealing temperature of 900 °C. The gases at the feed side were 20 mL min^−1^ H_2_ and 80 mL min^−1^ N_2_, while the sweep gas at permeate side was high purity Ar at a rate of ≈20 mL min^−1^. The electrical conductivity relaxation (ECR) technique, employing the four‐probe method, was utilized to measure the conductivity of the dense material bar in air and monitor the time required for the system to reach equilibrium upon varying the oxygen partial pressure. The single cell coated with Ag paste was outfitted with a connector specifically designed for electrochemical testing. The anode side of the cell was supplied with 25 mL min^−1^ wet hydrogen (3% H_2_O), while ambient air was supplied to the other electrode. An admiral electrochemical workstation was employed to measure the current and impedance values of the cell during the testing process.

### DFT Calculations

Density functional theory (DFT) calculations were carried out using the Vienna Ab initio simulation package (VASP) 5 with the projector‐augmented‐wave (PAW) method. The influences of Ni‐doping on the electronic state and the subsequent results were embodied in oxygen vacancy formation energy, hydration ability, and proton migrations. The convergence of energy and force of 10^−5^ eV and 0.02 eV Å^−1^ respectively were carried. A kinetic energy cutoff for a plane wave basis set of 400 eV was used and the K‐point mesh was set as a 4 × 4 × 4 gamma center grid. For optimization of the bulk structure, the 2 × 2 × 2 super cells were used for both BNFO and BFO. To make explorations for BNFO and BFO deeply at a theoretical level, the spin‐polarization method with generalized gradient approximation (GGA) and the Perdew‐Burke‐Ernzerhof (PBE) exchange‐correlation functional were utilized. Additionally, a climbing‐image nudged elastic band (CI‐NEB) method was performed to analyze the calculation of the reaction barriers of proton migration.

## Conflict of Interest

The authors declare no conflict of interest.

## Supporting information

Supporting Information

## Data Availability

The data that support the findings of this study are available from the corresponding author upon reasonable request.

## References

[1] a) L. Bi , S. P. Shafi , E. H. Da'as , E. Traversa , Small 2018, 14, e1801231; 29931743 10.1002/smll.201801231

[2] a) L. Bi , S. Boulfrad , E. Traversa , Chem. Soc. Rev. 2014, 43, 8255; 25134016 10.1039/c4cs00194j

[3] H. Ding , W. Wu , C. Jiang , Y. Ding , W. Bian , B. Hu , P. Singh , C. J. Orme , L. Wang , Y. Zhang , D. Ding , Nat. Commun. 2020, 11, 1907. 32312963 10.1038/s41467-020-15677-z PMC7171140

[4] a) Y. Zhang , B. Yu , S. Lü , X. Meng , X. Zhao , Y. Ji , Y. Wang , C. Fu , X. Liu , X. Li , Y. Sui , J. Lang , J. Yang , Electrochim. Acta 2014, 134, 107;

[5] a) Y. Xia , Z. Jin , H. Wang , Z. Gong , H. Lv , R. Peng , W. Liu , L. Bi , J. Mater. Chem. A 2019, 7, 16136;

[6] a) G. C. Mather , D. Muñoz‐Gil , J. Zamudio‐García , J. M. Porras‐Vázquez , D. Marrero‐López , D. Pérez‐Coll , Appl. Sci. 2021, 11, 5363;

[7] a) X. Zhu , Y. Cong , W. Yang , J. Membr. Sci. 2006, 283, 158

[8] Y. Chen , B. Qian , G. Yang , D. Chen , Z. Shao , J. Mater. Chem. A 2015, 3, 6501.

[9] F. Dong , D. Chen , Y. Chen , Q. Zhao , Z. Shao , J. Mater. Chem. 2012, 22, 15071.

[10] Z. Tao , L. Bi , Z. Zhu , W. Liu , J. Power Sources 2009, 194, 801.

[11] a) L. Bian , C. Duan , L. Wang , R. O'Hayre , J. Cheng , K.‐C. Chou , J. Mater. Chem. A 2017, 5, 15253;

[12] H. Tong , M. Fu , Y. Yang , F. Chen , Z. Tao , Adv. Funct. Mater. 2022, 32, 2209695.

[13] a) R. D. Shannon , Acta Cryst. 1976, 32, 751;

[14] J. Wang , K. Y. Lam , M. Saccoccio , Y. Gao , D. Chen , F. Ciucci , J. Power Sources 2016, 324, 224.

[15] Y. Wang , B. Hu , Z. Zhu , H. J. M. Bouwmeester , C. Xia , J. Mater. Chem. A 2014, 2, 136.

[16] L. Lei , Z. Tao , T. Hong , X. Wang , F. Chen , J. Power Sources 2018, 389, 1.

[17] D. Wang , Y. Xia , H. Lv , L. Miao , L. Bi , W. Liu , Int. J. Hydrogen Energy 2020, 45, 31017.

[18] J. J. Zhang , H. H. Wang , T. J. Zhao , K. X. Zhang , X. Wei , Z. D. Jiang , S. I. Hirano , X. H. Li , J. S. Chen , ChemSusChem 2017, 10, 2875. 28612461 10.1002/cssc.201700779

[19] a) T. Liu , H. Liu , X. Zhang , L. Lei , Y. Zhang , Z. Yuan , F. Chen , Y. Wang , J. Mater. Chem. A 2019, 7, 13550;

[20] Z. Tao , M. Fu , Y. Liu , Y. Gao , H. Tong , W. Hu , L. Lei , L. Bi , Int. J. Hydrogen Energy 2022, 47, 1947.

[21] X. Xu , H. Wang , J. Ma , W. Liu , X. Wang , M. Fronzi , L. Bi , J. Mater. Chem. A 2019, 7, 18792.

