# Supplementary material for: Nickel‐Regulated Composite Cathode with Balanced Triple Conductivity for Proton‐Conducting Solid Oxide Fuel Cells
Source: Adv Sci (Weinh). 2023 Oct 28;10(36):2304555. doi: 10.1002/advs.202304555 (PMC10754136; doi:10.1002/advs.202304555)
Supplement: Supplementary file 1 — Supporting Information [file ADVS-10-2304555-s001.pdf]

ADVANCED SCIENCE
Open Access

Supporting Information

for *Adv. Sci.*, DOI 10.1002/advs.202304555

Nickel-Regulated Composite Cathode with Balanced Triple Conductivity for Proton-Conducting Solid Oxide Fuel Cells

*Hua Tong, Wenjing Hu, Min Fu, Chunli Yang and Zetian Tao**

Supplementary information

Nickel-Regulated Composite Cathode with Balanced Triple Conductivity for Proton-conducting Solid Oxide Fuel Cells

Hua Tong[1], Wenjing Hu[1], Min Fu[1], Chunli Yang[2], Zetian Tao [1]*

1 School of Resources, Environment and Safety Engineering, University of South China, Hengyang, Hunan Province 421001, China

2 College of Materials Science and Engineering, Xi'an University of Architecture and Technology, Xi'an 710043, China

* Corresponding author.

Email address: newton@mail.ustc.edu.cn and taozetian@usc.edu.cn (Z. Tao).

1. Supplementary Figures

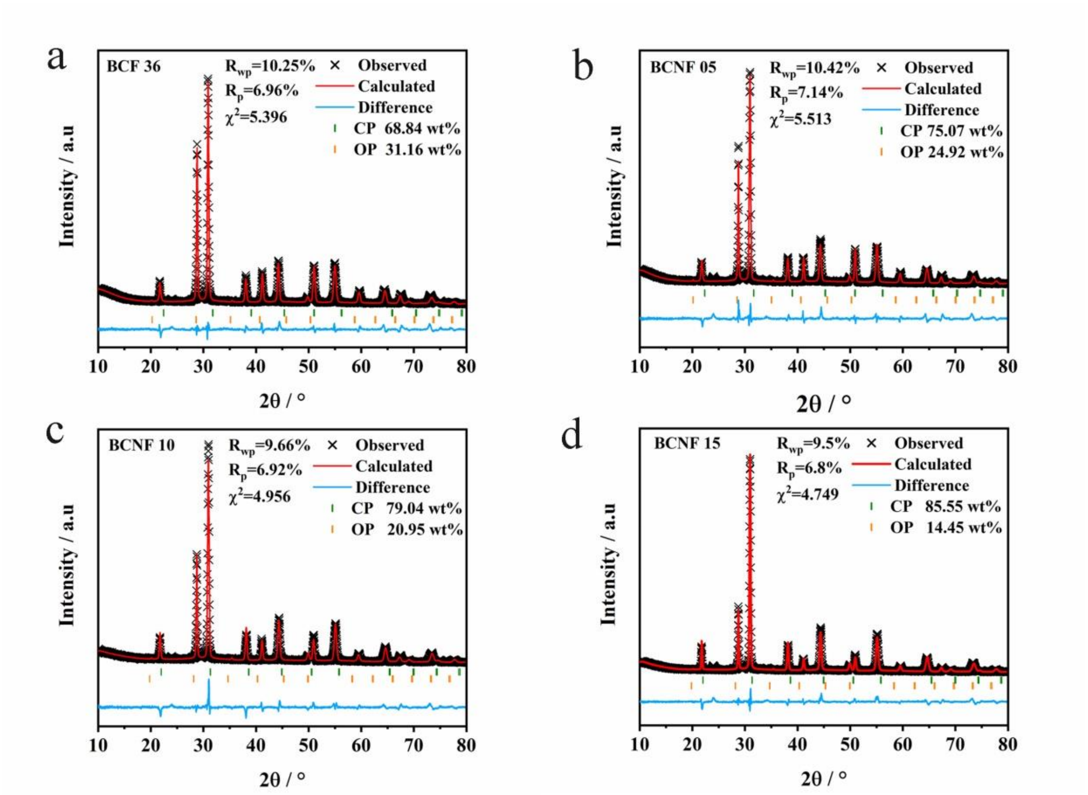

Figure S1. Refined XRD profiles of (**a**) $BaCe_{0.36}Fe_{0.64}O_{3-\delta}$ (BCF36), (**b**) $BaCe_{0.31}Ni_{0.05}Fe_{0.64}O_{3-\delta}$ (BCNF05), (**c**) $BaCe_{0.26}Ni_{0.10}Fe_{0.64}O_{3-\delta}$ (BCNF10) and (**d**)

$BaCe_{0.21}Ni_{0.15}Fe_{0.64}O_{3-\delta}$ (BCNF15) samples.

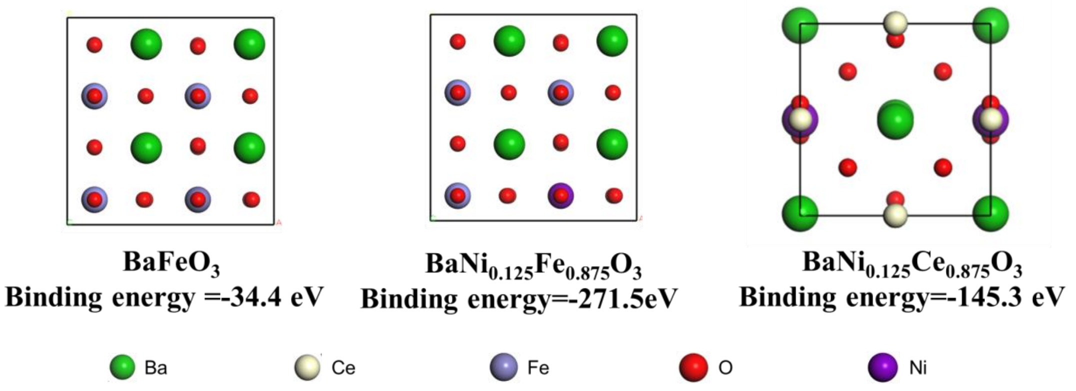

Figure S2. Binding energy for (**a**) $BaFeO_3$ (BFO), (**b**) $BaNi_{0.125}Fe_{0.875}O_3$ (BNFO) and (**c**) $BaNi_{0.125}Ce_{0.875}O_3$ (BNCO).

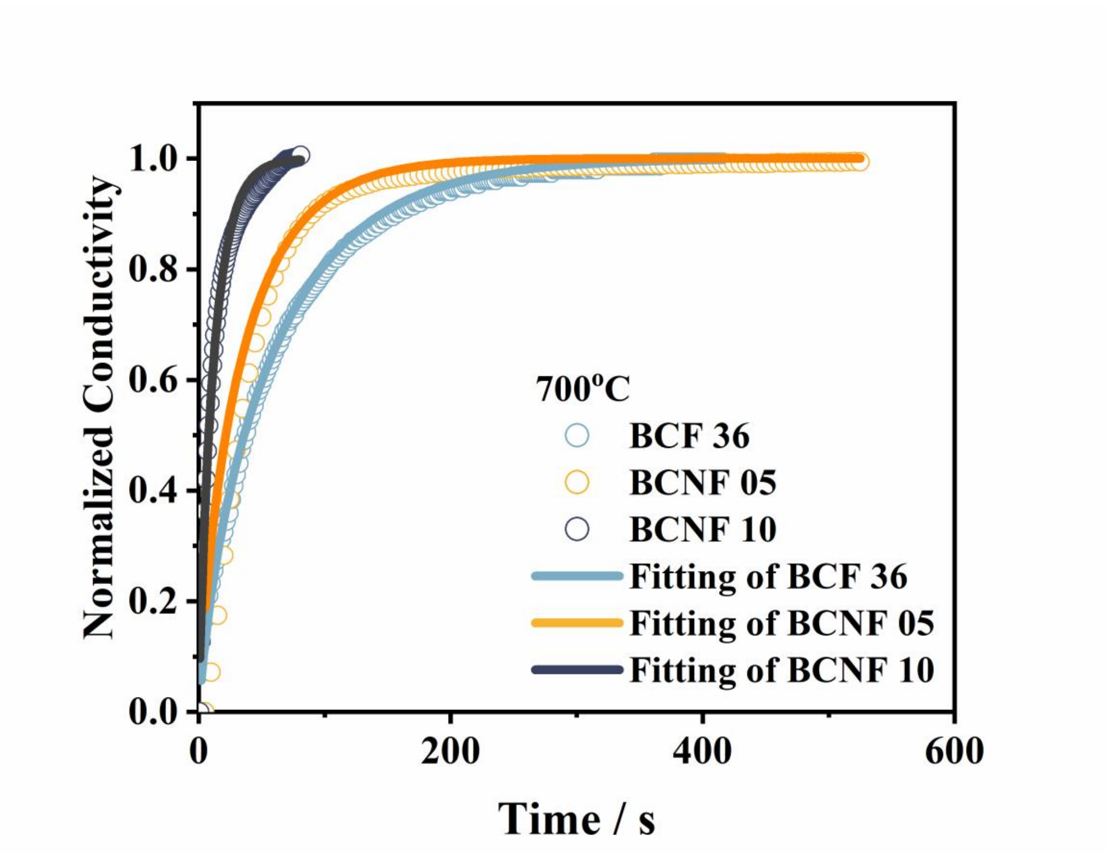

Figure S3. ECR curves of BCF36，BCNF05 and BCNF10 at 700℃ from dry air to O_2.

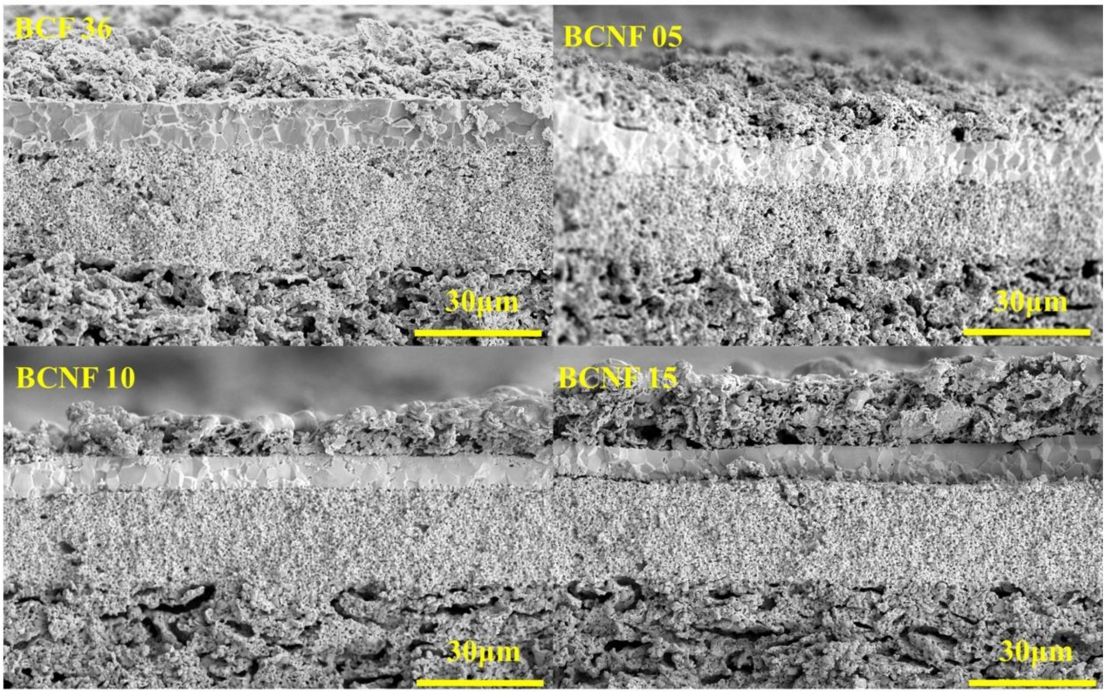

Figure S4. SEM images of cell cross-section for BCNFx.

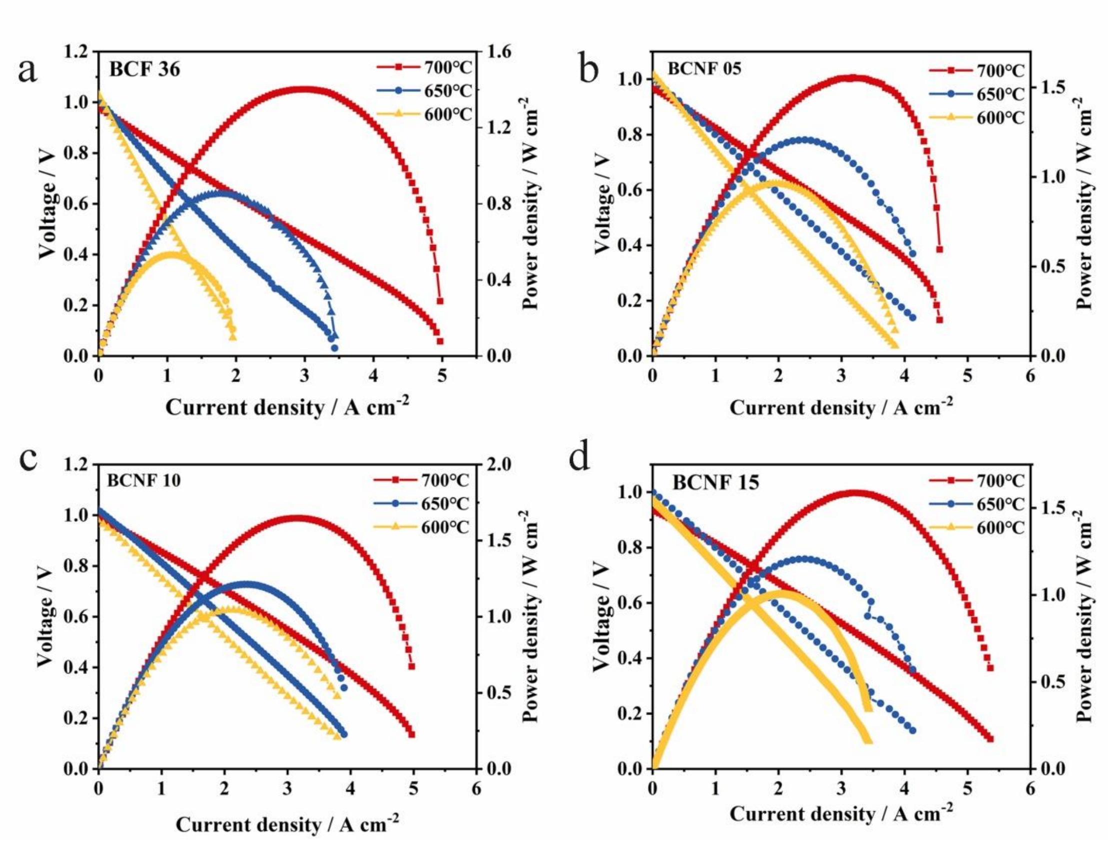

Figure S5. I–V curves and power densities of anode-supported single cell with cathode (**a**) BCF36, (**b**) BCNF05, (**c**) BCNF10 and (**d**) BCNF15.

.

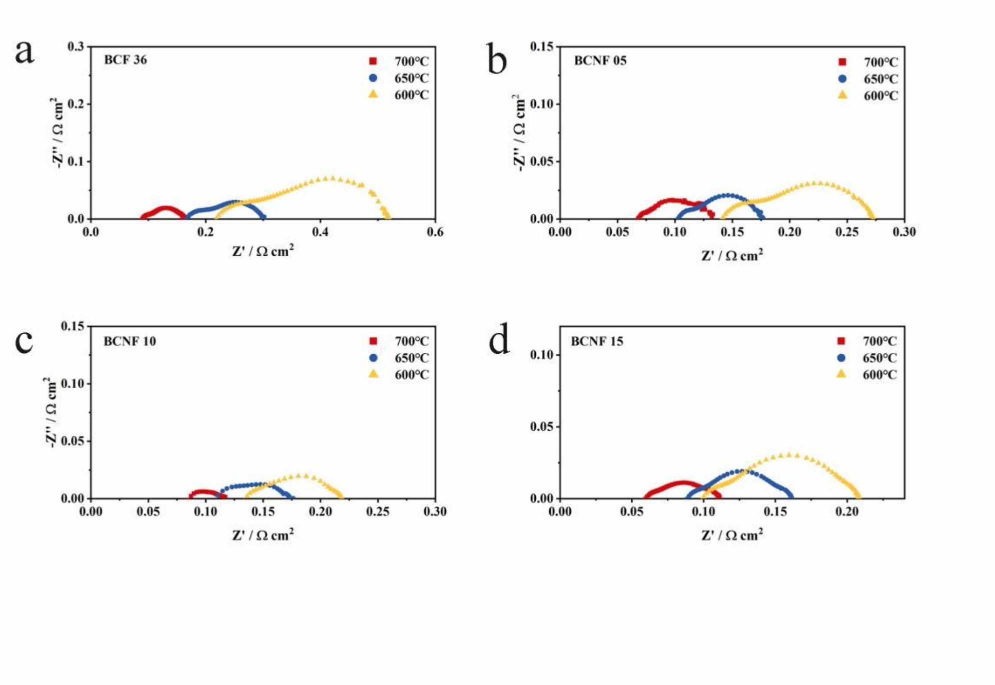

Figure S6. EIS curves of anode-supported single cell with cathode (**a**) BCF36, (**b**) BCNF05, (**c**) BCNF10 and (**d**) BCNF15.

.

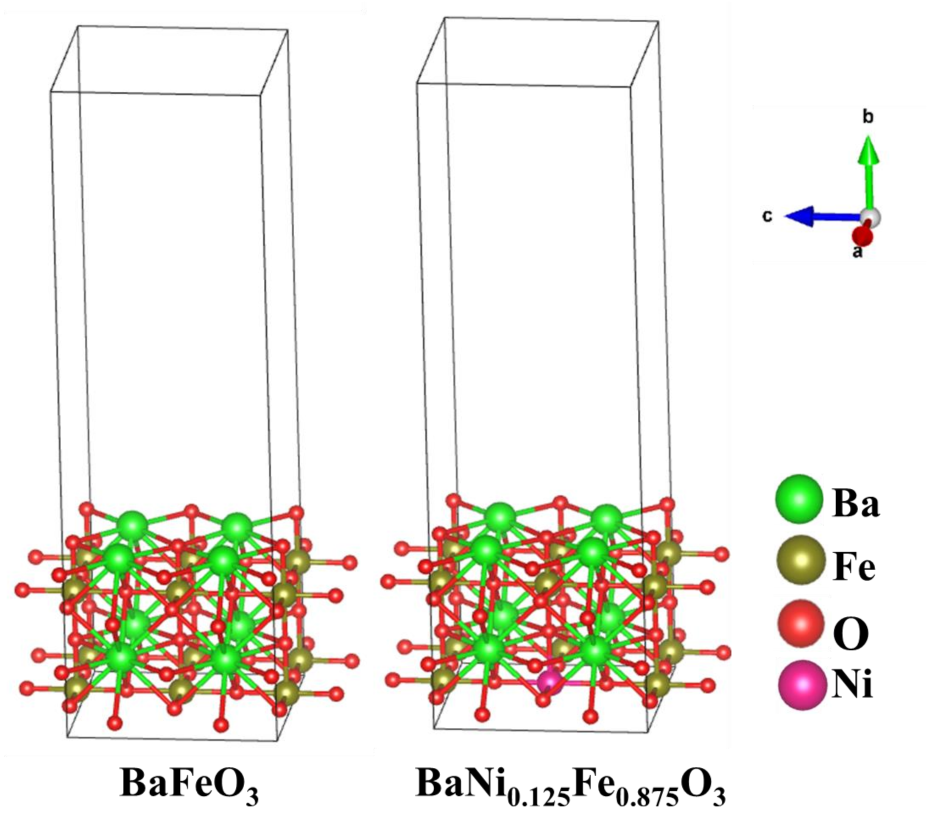

Figure S7. 2 × 2 × 2 supercell and vacuum layer of BFO and BNFO after optimize.

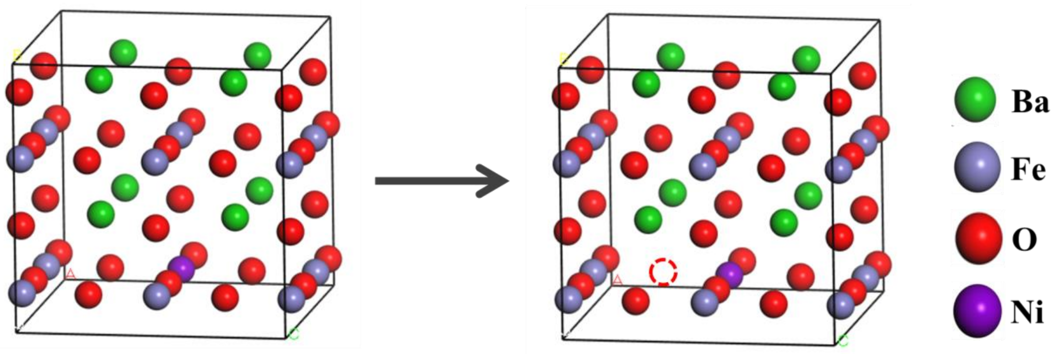

Figure S8. The oxygen with red dashed circle stand for the position where oxygen vacancies ($V_O^{\cdot\cdot}$) are created.

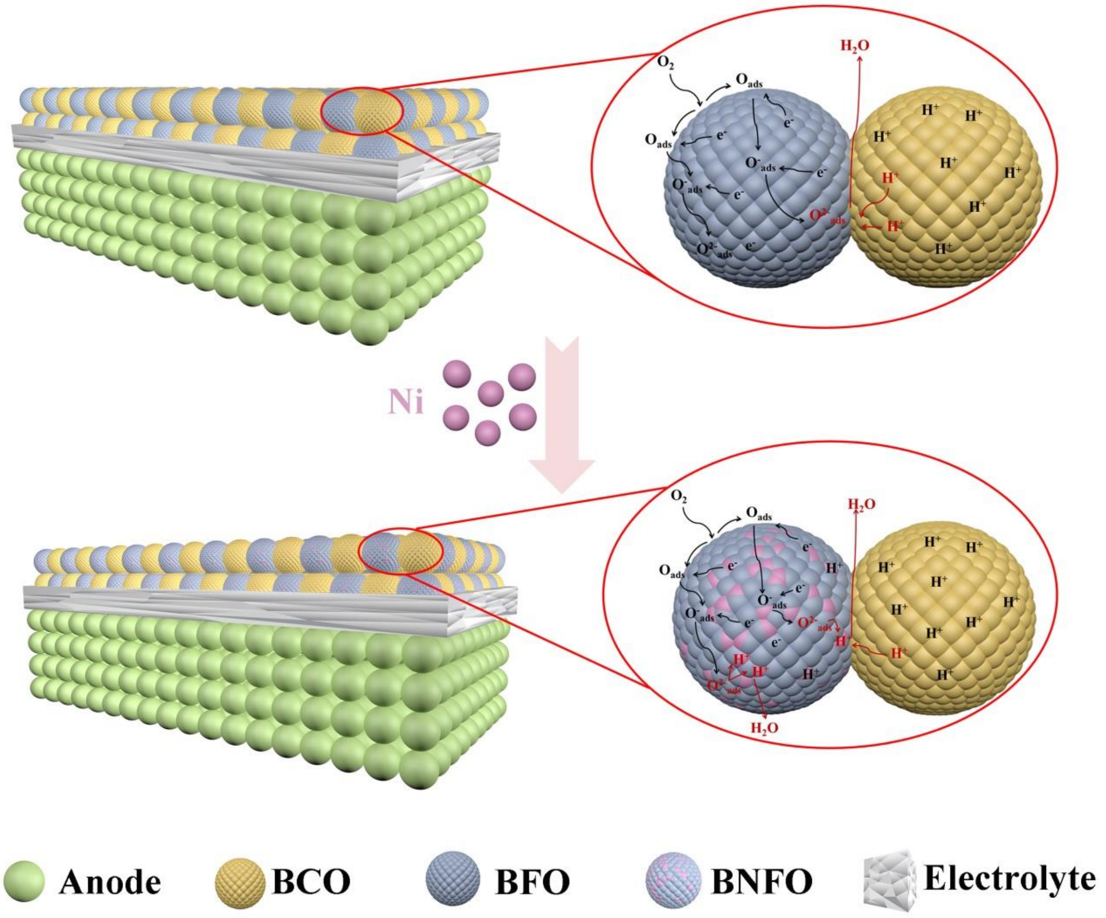

Figure S9. Schematic diagram of the ORR mechanism and three-phase conducting pathways of BCF36 and BCNF10.

2. Supplementary Tables

Supplementary Table 1. Cell parameters of the BCNFx (x=0, 0.05, 0.1, 0.15) samples.

	x=0	x=0.05	x=0.1	x=0.15
Lattice parameters				
a/Å	4.092751781	4.081788099	4.080759362	4.080116675
b/Å	4.092751781	4.081788099	4.080759362	4.080116675
c/Å	4.092751781	4.081788099	4.080759362	4.080116675
V/Å^3	68.55612	68.00665	67.95524	67.92314

Supplementary Table 2. D, K values from ECR curve fitting for BCNFx.

		600℃	650℃	700℃
BCF 36	D(cm^2 s^{-1})	3.8e-5	2.0e-4	3.0e-4
	K(cm s^{-1})	8.0e-4	1.2e-3	5.0e-3
BCNF 05	D(cm^2 s^{-1})	2.0e-4	3.5e-4	3.7e-4
	K(cm s^{-1})	4.0e-3	6.5e-3	6.8e-3
BCNF 10	D(cm^2 s^{-1})	7.5e-4	7.5e-4	8.5e-4
	K(cm s^{-1})	9.0e-3	9.5e-3	9.5e-3

Supplementary Table 3. Molar ratio between adsorbed oxygen to lattice oxygen for BCNFx.

	Oads	Olat	Oads/Olat
BCF 36	87.65%	12.35%	7.097
BCNF 05	89.7%	10.3%	8.7
BCNF 10	94.7%	5.3%	17.86
BCNF 15	94.48%	5.52%	17.1

Supplementary Table 4. Performance comparison of SOFCs reported in the literature and in this study with different $BaFeO_3$-based cathode materials measured at 700 °C and 600℃

ANODE	ELECTROLYTE	CATHODE	Rp		MPDs		REF
			700	600	700	600	

NiO- BZCY	BZCY	BCFB	0.098	0.445	736	362	[1]
NiO-SDC	SDC	BCF	0.046	0.186	415	212	[2]
NiO-BZCY	BZCY	BFZB	0.055	0.21	816.26	510	[3]
NiO-BZCY	BZCY	BFCY-BCFY	0.05	0.25	750	417	[4]
NiO-BCZYYb3511	BCZYYb3511	BLFZ	/	0.08	1160	780	[5]
NiO-BZCYYb	BZCYYb	BCYF2	/	0.18	/	656	[6]
NiO-BZCY	BZCY	BSF-SDC	0.192	0.563	440 ± 8	182 ± 13	[7]
NiO-BZCY	BZCY	PBF-SDC	0.277	0.921	332 ± 20	160 ± 2	[7]
NiO-BZCY	BZCY	PBSF-SDC	0.173	0.912	456 ± 50	153 ± 11	[7]
NiO-BCZYYb	BCZYYb	BLFNi0.2-BCZYYb	0.099	0.251	631	241	[8]
NiO–YSZ	thin-film YSZ electrolyte, SDC buffer layer	BLF	0.037	0.211	1125	610	[9]
NiO-BZCY	BZCY	BCF36	0.060	0.131	1056	440	[10]
NiO-BZCY	BZCY	BCF50	0.17	0.8	395	190	[11]
NiO-BZCY	BZCY	BCFP0.2	0.057	/	562	316	[12]
NiO-BZCY	BZCY	BZFN15-BZCY	0.087	0.443	583	300	[13]
NiO-BZCYYb	BZCYYb	BLFZ–BZCYYb	0.159	0.683	266.5	142.8	[14]
NiO-BZCY	BZCY	BFSBi0.3	0.032	0.094	1277	841	[15]
NiO-BZCY	BZCY	BCNF10	0.028	0.082	1647	1044	This work

Notes:
BCFB=$BaCe_{0.5}Fe_{0.3}Bi_{0.2}O_{3-\delta}$; SDC=$Sm_{0.2}Ce_{0.8}O_{1.9}$; BCF=$BaCe_{0.05}Fe_{0.95}O_{3-\delta}$; BZCY=$BaZr_{0.1}Ce_{0.7}Y_{0.2}O_{3-\delta}$; BCZYYb3511=$Ba(Ce_{0.3}Zr_{0.5}Y_{0.1}Yb_{0.1})O_{3-\delta}$; BFZB=$BaFe_{0.8}Zn_{0.1}Bi_{0.1}O_{3-\delta}$; BCFY=$BaCe_{0.7}Fe_{0.1}Y_{0.2}O_{3-\delta}$; BFCY=$BaFe_{0.8}Ce_{0.1}Y_{0.1}O_{3-\delta}$; BLFZ=$(Ba_{0.95}La_{0.05})(Fe_{0.8}Zn_{0.2})O_{3-\delta}$; BCZYYb=$BaCe_{0.7}Zr_{0.1}Y_{0.1}Yb_{0.1}O_{3-\delta}$; BCYF2=$BaCe_{0.16}Y_{0.04}Fe_{0.8}O_{3-\delta}$; BSF=$Ba_{0.5}Sr_{0.5}FeO_{3-\delta}$; PBF=$Pr_{0.5}Ba_{0.5}FeO_{3-\delta}$; PBSF=$Pr_{0.5}Ba_{0.25}Sr_{0.25}FeO_{3-\delta}$; BLFNi0.2=$Ba_{0.95}La_{0.05}Fe_{0.8}Ni_{0.2}O_{3-\delta}$; BLF=$Ba_{0.95}La_{0.05}FeO_{3-\delta}$; BCF36=$BaCe_{0.36}Fe_{0.64}O_{3-\delta}$; BCF50=$BaCe_{0.5}Fe_{0.5}O_{3-\delta}$; BCFP0.2=$BaCe_{0.2}Fe_{0.6}Pr_{0.2}O_{3-\delta}$; BZFN15=$BaZr_{0.1}Fe_{0.75}Ni_{0.15}O_{3-\delta}$; BLFZ=$Ba_{0.95}La_{0.05}Fe_{0.8}Zn_{0.2}O_{3-\delta}$; BFSBi0.3=$BaFe_{0.5}Sn_{0.2}Bi_{0.3}O_{3-\delta}$; BCNF10=$BaCe_{0.26}Ni_{0.1}Fe_{0.64}O_{3}$

3. Supplementary References

[1] D. Shan, Z. Gong, Y. Wu, L. Miao, K. Dong, W. Liu, *Ceramics International* **2017**, *43* (4), 3660, https://doi.org/10.1016/j.ceramint.2016.11.206.

[2] H. Liu, K. Zhu, Y. Liu, W. Li, L. Cai, X. Zhu, M. Cheng, W. Yang, *Electrochimica Acta* **2018**, *279*, 224, https://doi.org/10.1016/j.electacta.2018.05.086.

[3] Y. Xia, X. Xu, Y. Teng, H. Lv, Z. Jin, D. Wang, R. Peng, W. Liu, *Ceramics International* **2020**, *46* (16), 25453, https://doi.org/10.1016/j.ceramint.2020.07.015.

[4] Z. Wei, J. Wang, X. Yu, Z. Li, Y. Zhao, J. Chai, *International Journal of Hydrogen Energy* **2021**, *46* (46), 23868, https://doi.org/10.1016/j.ijhydene.2021.04.188.

[5] S. K. Kim, S. H. Hwang, J.-T. Nam, J.-S. Park, *Journal of Power Sources* **2021**, *513*, 230544, https://doi.org/10.1016/j.jpowsour.2021.230544.

[6] D. Zou, Y. Yi, Y. Song, D. Guan, M. Xu, R. Ran, W. Wang, W. Zhou, Z. Shao, *Journal of Materials Chemistry A* **2022**, *10* (10), 5381, https://doi.org/10.1039/d1ta10652j.

[7] B. Cai, T.-F. Song, J.-R. Su, H. He, Y. Liu, *Solid State Ionics* **2020**, *353*, 115379, https://doi.org/10.1016/j.ssi.2020.115379.

[8] J. Jing, Z. Lei, Z. Wu, Z. Wang, H. Yu, Z. Yang, S. Peng, *Journal of the European Ceramic Society* **2022**, *42* (14), 6566, https://doi.org/10.1016/j.jeurceramsoc.2022.07.003.

[9] F. Dong, D. Chen, Y. Chen, Q. Zhao, Z. Shao, *Journal of Materials Chemistry* **2012**, *22* (30), 15071, https://doi.org/10.1039/c2jm31711g.

[10] H. Tong, M. Fu, Y. Yang, F. Chen, Z. Tao, *Advanced Functional Materials* **2022**, *32*, 2209695.

[11] Z. Tao, L. Bi, Z. Zhu, W. Liu, *Journal of Power Sources* **2009**, *194* (2), 801, https://doi.org/10.1016/j.jpowsour.2009.06.071.

[12] X. Zhou, N. Hou, T. Gan, L. Fan, Y. Zhang, J. Li, G. Gao, Y. Zhao, Y. Li, *Journal of Power Sources* **2021**, *495*, 229776, https://doi.org/10.1016/j.jpowsour.2021.229776.

[13] J. Wang, Z. Li, H. Zang, Y. Sun, Y. Zhao, Z. Wang, Z. Zhu, Z. Wei, Q. Zheng, *International Journal of Hydrogen Energy* **2022**, *47* (15), 9395, https://doi.org/10.1016/j.ijhydene.2022.01.012.

[14] Z. Wang, P. Lv, L. Yang, R. Guan, J. Jiang, F. Jin, T. He, *Ceramics International* **2020**, *46* (11), 18216, https://doi.org/10.1016/j.ceramint.2020.04.144.

[15] Y. Xia, Z. Jin, H. Wang, Z. Gong, H. Lv, R. Peng, W. Liu, L. Bi, *Journal of Materials Chemistry A* **2019**, *7* (27), 16136, https://doi.org/10.1039/c9ta02449b.
